# Mas-Related G-Protein-Coupled Receptors: Emerging Roles in Neuropathic Pain

**DOI:** 10.3390/biom16020290

**Published:** 2026-02-12

**Authors:** Mario García-Domínguez

**Affiliations:** 1 Program of Immunology and Immunotherapy, CIMA-Universidad de Navarra, 31008 Pamplona, Spain; mgdom@unav.es; 2 Department of Immunology and Immunotherapy, Clínica Universidad de Navarra, 31008 Pamplona, Spain; 3 Centro de Investigación Biomédica en Red de Cáncer (CIBERONC), 28029 Madrid, Spain

**Keywords:** neuropathic pain, neuroimmune signaling, GPCRs, ion channels, hyperexcitability, analgesic targets

## Abstract

Mas-related G-protein-coupled receptors constitute a distinct family of GPCRs expressed in some subsets of sensory neurons and immune cells. Increasing evidence highlights their contribution to the modulation of nociceptive signaling and neuroimmune interactions. Recent studies demonstrate that Mas-related G-protein-coupled receptors are implicated not only in itch transmission but also in the pathophysiology of neuropathic pain, where aberrant receptor activity influences neuronal excitability, glial activation, and inflammatory responses. This review summarizes current knowledge on the molecular mechanisms by which Mas-related G-protein-coupled receptors regulate pain hypersensitivity, including their interactions with ion channels, neuropeptides, and immune mediators. Moreover, the potential of targeting specific Mas-related G-protein-coupled receptor subtypes for therapeutic intervention is discussed, emphasizing their promise as novel druggable candidates for neuropathic pain, the emerging management. Clarifying the roles of Mas-related G-protein-coupled receptors in sensory modulation may provide critical insights into the development of mechanism-based analgesics.

## 1. Introduction

According to the International Association for the Study of Pain (IASP), pain is defined as “an unpleasant sensory and emotional experience associated with, or resembling that associated with, actual or potential tissue damage” [1,2]. Pain is highly characterized as a multifactorial and inherently multidimensional phenomenon, acting as a critical diagnostic marker and encompassing sensory-discriminative, affective-emotional, and cognitive-evaluative dimensions, frequently indicating underlying health conditions that necessitate medical evaluation [3,4]. Pain can be divided into three principal types according to the causative agent. Nociceptive pain arises from real or potential damage to non-neural tissue through nociceptor activation [5], whereas nociplastic pain results from altered nociception without evident tissue injury or detectable somatosensory lesions [6].

Neuropathic pain is a chronic, prevalent, and disabling condition affecting approximately 7–10% of the population [7,8,9]. Its etiology is highly heterogeneous, encompassing traumatic nerve injury, chemotherapy-induced neuropathy (CIPN), numerous metabolic disorders (e.g., diabetes), viral infections (e.g., HIV/AIDS or herpes zoster), autoimmune neuropathies (e.g., Guillain-Barré syndrome -GBS-), spinal cord injury, and neurodegenerative diseases (e.g., multiple sclerosis) [10,11,12,13,14,15,16]. Clinically, neuropathic pain manifests as a constellation of positive (e.g., spontaneous burning pain and electric-shock-like sensations) and negative (e.g., loss of sensory function) symptoms, as well as evoked phenomena such as allodynia (pain in response to non-noxious stimuli) and hyperalgesia (exaggerated pain to noxious stimuli) [17]. Despite this heterogeneity, a defining feature of neuropathic pain is persistent neuroplasticity across numerous levels of the nociceptive pathway, encompassing peripheral sensitization, ectopic activity in injured sensory neurons, maladaptive neuron-immune interactions, synaptic remodeling in the spinal dorsal horn, and dysregulated descending pain modulation [18,19,20]. In terms of molecular pathways, peripheral or central injury triggers the release of numerous pro-inflammatory cytokines (e.g., TNF-α, IL-1β, and IL-6), chemokines (e.g., CCL2, CCL3, and CCL5), and damage-associated molecular patterns (DAMPs) [21,22,23]. Moreover, activated glial cells further release pro-inflammatory mediators and neurotrophic factors (e.g., IL-1β, TNF-α, BDNF, and NGF), while chemokine gradients recruit peripheral immune cells to injured nerves and central pain-processing regions [24,25]. These processes induce alterations in several ion channels (e.g., VGSCs, VGCCs, and K^+^ channels) and neurotransmitter systems, thus promoting neuronal hyperexcitability [26,27,28].

Current therapeutic strategies remain limited in both efficacy and tolerability. First-line pharmacotherapies (such as gabapentinoids, tricyclic antidepressants -TCAs-, serotonin-noradrenaline reuptake inhibitors -SNRIs-, and numerous sodium channel blockers) produce clinically relevant pain reduction in approximately 30% of patients [29,30]. Opioids show reduced long-term effectiveness in neuropathic conditions and introduce risks of dependence, tolerance, respiratory depression, and opioid-induced hyperalgesia [31]. Even emerging approaches such as monoclonal antibodies or neurostimulation have not fully addressed the critical need for mechanism-based, peripherally selective analgesics [32,33]. This therapeutic stagnation highlights the need to identify novel molecular targets capable of modulating pathological pain signals without disrupting normal nociceptive function or causing systemic side effects.

One such group of targets is the family of G-protein-coupled receptors (GPCRs), the largest and pharmacologically most exploited receptor class in the human genome [34]. GPCRs act as molecular transducers that convert extracellular chemical signals into intracellular second-messenger cascades, thus regulating neuronal excitability, synaptic plasticity, neurotransmitter release, and neuroimmune interactions [35]. Many of the most effective analgesic drugs currently in clinical use (including μ-opioid receptor agonists, cannabinoid receptor agonists, and α2-adrenergic receptor ligands) achieve their actions via GPCR signaling pathways, demonstrating the druggability of this receptor family [36].

Within this superfamily, the Mas-related G-protein-coupled receptors (Mrgprs) have emerged as a particularly compelling subset due to their restricted expression in small-diameter nociceptors of dorsal root (DRG) and trigeminal ganglia and their unique pharmacological profiles [37,38]. Firstly, classified as orphan receptors, Mrgprs are now known to respond to a diverse spectrum of ligands, like endogenous amino acids and neuropeptides (e.g., BAM8-22, β-alanine, and substance P), inflammatory mediators, and some exogenous xenobiotics [39,40]. Functionally, several Mrgpr subtypes (principally MrgprA3, MrgprC11, and the human ortholog MRGPRX1) have been implicated in both pruriceptive and nociceptive signaling [41,42,43,44,45]. Their activation initiates canonical Gαq/11-phospholipase C (PLC) pathways, leading to IP_3_ formation, intracellular Ca^2+^ mobilization, and downstream protein kinase C (PKC)-mediated sensitization of transient receptor potential (TRP) channels, like TRPA1 and TRPV1 [45,46,47]. Alongside neuronal activation, Mrgprs participate in bidirectional mast cell-neuron signaling, modulate cytokine and neuropeptide release, and influence glial reactivity in the dorsal horn during chronic pain [48,49,50,51].

Current evidence highlights Mrgprs as fundamental molecular nodes connecting peripheral damage, inflammatory signaling, and neuronal hyperexcitability, underlying the chronicity of neuropathic pain [52,53]. Their distribution, ligand diversity, and mechanistic involvement in pathological but not baseline nociception make them a promising, yet still underexploited, class of therapeutic targets [54]. A more comprehensive understanding of Mrgpr signaling might enable the development of next-generation analgesics with improved selectivity, reduced central side effects, and minimal addiction liability [50].

This review synthesizes current knowledge of Mrgprs and their key roles in the pathophysiology of neuropathic pain, a condition whose high prevalence and limited responsiveness to existing therapies make it an ideal context for investigating Mrgprs as potential targets for next-generation, mechanism-based analgesics. The molecular mechanisms by which Mrgprs modulate nociceptor excitability, neuropeptide release, and neuroimmune communication are examined, highlighting their contribution to persistent pain and hypersensitivity. Preclinical and translational evidence support Mrgprs as promising targets for next-generation analgesics.

## 2. The Mas-Related G-Protein-Coupled Receptor Family

### 2.1. Mrgpr Classification and Subtype Diversity

Mrgprs represent a discrete subfamily of class A GPCRs distinguished by extensive evolutionary diversification and a predominantly peripheral expression pattern [55]. In contrast to canonical GPCR families that exhibit high cross-species sequence conservation, the Mrgpr repertoire displays substantial lineage-specific expansion, mainly in rodents, suggesting adaptive evolution driven by pressures in sensory and host-defense pathways [56]. Mrgprs were initially identified through transcriptomic analyses of DRG, where they were characterized as orphan GPCRs selectively expressed in subsets of nociceptive and pruriceptive neurons [57,58]. Since then, this family has expanded to include many receptors with divergent ligand selectivity, tissue distribution, and physiological roles [50].

In mice, the Mrgpr gene family is represented by more than 50 genes, many located in two large clusters on chromosome 7 [50]. These receptors are classified into the Mrgpra, Mrgprb, Mrgprc, Mrgprd, Mrgpre, Mrgprf, Mrgprg, and Mrgprx subfamilies (Table 1), although the boundaries between subgroups are based primarily on sequence similarity rather than strict phylogenetic lineage [50], and several murine receptors lack direct human orthologs, complicating translational research by limiting direct cross-species extrapolation of pharmacological findings [39]. The human genome, in contrast, contains a very reduced number of functional Mrgpr genes, such as MRGPRX1-4, MRGPRD, MRGPRE, MRGPRF, and a small number of additional loci (Table 1) [40], and this contraction of the gene family in primates is not indicative of functional loss but rather reflects a shift toward receptor specialization instead of multiplicity [55].

Overall, Mrgpr classification is an evolving framework informed by comparative genomics, structural studies, and functional de-orphanization. Once considered a niche sensory receptor group, Mrgprs are now recognized as key molecular mediators at the neuro-immune interface, with ongoing single-cell transcriptomics, humanized mouse models, and structure-guided ligand discovery poised to further refine their taxonomy and functional roles [46,59,60].

### 2.2. Molecular Signaling Pathways

A key molecular characteristic of Mrgprs is their divergence from several highly conserved motifs present in canonical class A GPCRs. Unlike prototypical class A receptors, many Mrgpr subtypes, such as MrgprA3, MrgprC11, and MrgprD, do not reserve the canonical DRY motif (Asp-Arg-Tyr) at the cytoplasmic end of TM3 or the NPxxY motif (Asp-Pro-x-x-Tyr) on TM7, motifs that function as microswitches for activation and G protein coupling, rather than directly defining the orthosteric binding site [39,46].

Independent of these microswitch motifs, sequence comparisons across Mrgpr subfamilies also reveal reduced conservation of several residues that conventionally constitute the orthosteric binding pocket [46]. In Mrgprs, these positions frequently exhibit substitutions to residues with divergent side chains (e.g., non-polar or small aliphatic residues in place of conserved polar/aromatic residues), and the overall pattern of side-chain chemical landscape of the pocket is heterogeneous across the MrgprA, MrgprB, and MrgprC clades [46,61,62].

**Figure 1:**
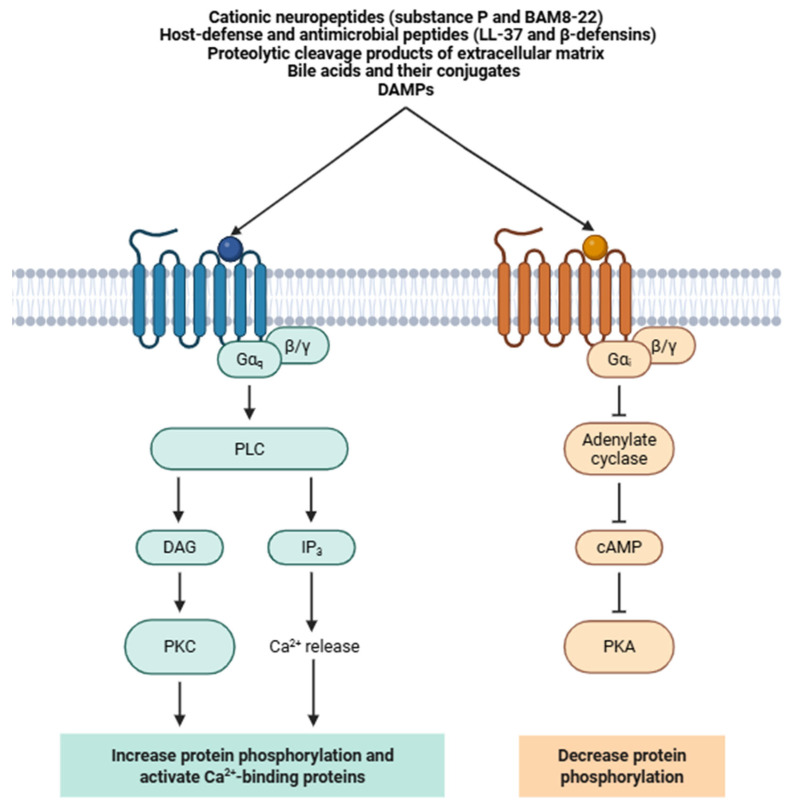
Numerous neuropeptides (e.g., cationic neuropeptides and host-defense peptides) activate Mrgprs, driving several signaling pathways (PLC-DAG-IP_3_ and cAMP-PKA) to modulate inflammatory responses, sensory cell activation, and neuroimmune communication. Abbreviations: PLC (phospholipase C), PKC (protein kinase C), PKA (protein kinase A), GPCR (G-protein-coupled receptor), DAG (diacylglycerol), IP_3_ (inositol 1,4,5-trisphosphate), cAMP (cyclic adenosine monophosphate), Ca^2+^ (calcium ion), Gαi (G alpha i subunit), Gαq (G alpha q subunit), Gβγ (G beta-gamma subunit), DAMP (damage-associated molecular pattern), BAM8-22 (bovine adrenal medulla peptide 8–22), and LL-37 (cathelicidin antimicrobial peptide LL-37).

Endogenous agonists of Mrgprs encompass an exceptionally diverse array of molecular species (Figure 1), including cationic neuropeptides (e.g., substance P and BAM8-22) [63], host-defense and antimicrobial peptides (e.g., LL-37 and β-defensins) [64], proteolytic cleavage products of extracellular matrix [65], bile acids and their conjugates [66], and DAMPs [55]. Exogenous ligands include a wide range of structurally unrelated small molecules, including anesthetics [67], neuromodulatory drugs [68], opioids [68], and environmental irritants [69], many of which act as biased agonists or partial agonists depending on the receptor subtype and cellular context. A subset of Mrgpr paralogs remain orphan receptors, whose cognate ligands have yet to be identified [70].

Finally, while this structural divergence in the putative ligand-facing residues is consistent with functional studies showing that different Mrgpr subtypes can be activated by chemically distinct ligands, direct biophysical evidence linking these specific residue differences to broad “promiscuity” in ligand binding is currently limited. Consequently, rather than describing Mrgprs as inherently promiscuous, a more precise interpretation is that divergence from conserved orthosteric pocket residues contributes to the diversification of ligand sensitivity profiles across Mrgpr subfamilies, in a ligand class- and receptor subtype-dependent manner [61].

At the signaling level, most members of the Mrgpr family preferentially couple to the Gαq/11 subfamily of heterotrimeric G proteins (Figure 1) [71]. Upon agonist engagement, conformational rearrangements within the receptor (principally rotational and outward movements of transmembrane helices 5, 6, and 7) stabilize an active-state conformation that evokes GDP-GTP exchange on the Gαq/11 subunit [44]. This nucleotide exchange triggers the dissociation of the Gαq/11-GTP complex from the Gβγ dimer, allowing both signaling entities to interact with distinct downstream effectors. Activated Gαq/11 subsequently engages and activates phospholipase Cβ (PLCβ), which hydrolyzes the membrane phospholipid phosphatidylinositol 4,5-bisphosphate (PIP_2_) into two second messengers: inositol 1,4,5-triphosphate (IP_3_) and diacylglycerol (DAG) [72]. IP_3_ diffuses via the cytosol and binds to many IP_3_ receptors (IP_3_Rs) located on the endoplasmic reticulum membrane, inducing Ca^2+^ efflux from the endoplasmic reticulum into the cytoplasm [73]. The resulting increase in Ca_i_^2+^ triggers various downstream processes, including activation of Ca^2+^-dependent cation channels (TRPA1 and TRPV1), membrane depolarization, and neurotransmitter or neuropeptide release (e.g., substance P and calcitonin gene-related peptide -CGRP-) [48].

Beyond canonical Gαq/11 coupling, several Mrgpr subtypes exhibit coupling promiscuity or context-dependent signaling plasticity. Depending on receptor subtype, ligand bias, and cellular milieu, some Mrgprs can alternatively engage Gαi/o proteins or β-arrestin-dependent scaffolding pathways [55]. Coupling to Gαi/o can lead to inhibition of adenylyl cyclase, resulting in reduced cyclic AMP (cAMP) levels and modulation of protein kinase A (PKA)-dependent signaling [72]. In contrast, β-arrestin recruitment not only contributes to receptor internalization and desensitization but can also scaffold mitogen-activated protein kinase (MAPK) cascades, including ERK1/2 and p38 MAPK pathways [74]. β-Arrestin-mediated signaling, as observed for MRGPRX2 and MRGPRX4 in mast cells and dependent on β-arrestins 1 and 2, is usually spatially and temporally distinct from G protein-dependent pathways, potentially giving rise to compartmentalized downstream effects [75,76,77].

### 2.3. Characterized Physiological and Pathophysiological Actions of Mrgprs

Rapid advances in single-cell transcriptomics, in vivo Ca^2+^ imaging, ligand screening technologies, and structural biology have expanded the conceptual framework of Mrgpr biology [78,79]. It is now evident that Mrgprs are not only determinants of sensory neuron identity but also central nodes in intercellular communication between neurons, immune and epithelial cells, and microbiota-derived environmental signals [80,81,82]. Their biological diversity reflects both evolutionary pressures and molecular specialization, resulting in unique patterns of expression, ligand selectivity, and intracellular signaling architecture unparalleled among GPCR families [37,38].

In sensory neurons, Mrgprs (e.g., MrgprA3, MrgprC11, and MrgprD) define distinct pruriceptive and polymodal sensory populations with unique molecular signatures and central projection patterns [47,72,83,84]. MrgprA3 marks a genetically defined lineage that constitutes one of the principal pathways for non-histaminergic itch, responding robustly to chloroquine, SLIGRL peptides, and potentially endogenous pruritogenic metabolites generated under pro-inflammatory conditions [83,84,85]. MrgprC11 shows a broader ligand range, responding to proenkephalin-derived peptides (e.g., BAM8-22), as well as neuropeptides and cytokine-associated factors released during tissue stress [86,87]. MrgprD labels a separate mechanosensitive population implicated in punctate mechanical nociception, environmental toxin detection, and metabolic chemosensation [88,89].

On the other hand, pathological dysregulation of Mrgprs contributes to chronic pruritic, inflammatory, and hypersensitivity conditions [47]. Overactivity of MrgprA3- and MrgprC11-positive neurons is a defining feature of atopic dermatitis, prurigo nodularis, and systemic pruritus [90,91]. These neurons experience transcriptional reprogramming during chronic inflammation, leading to increased receptor expression, heightened excitability, and enhanced responsiveness to pruritogenic mediators [47]. The resulting feed-forward neuroimmune loops perpetuate persistent itch, scratching-induced barrier disruption, and secondary infections that exacerbate disease severity [92].

In non-neuronal tissues, Mrgprs serve roles of comparable significance. MRGPRX2, prominently expressed on human skin- and tissue-resident mast cells, operates as a crucial hub for IgE-independent mast-cell activation [93,94,95]. Its ability to detect several cationic amphipathic peptides (e.g., LL-37, β-defensins, substance P, neurotensin, and vasoactive intestinal peptide) allows it to integrate neural, epithelial, and microbial signals into coordinated inflammatory responses [96,97]. Some small-molecule drugs such as fluoroquinolones, vancomycin, icatibant, neuromuscular-blocking agents, and certain opioids activate MRGPRX2, producing degranulation and mediator release without requirement for prior sensitization [98]. This mechanism underlies a substantial proportion of pseudo-allergic drug reactions characterized by flushing, urticaria, angioedema, bronchospasm, and anaphylactoid events [99]. MRGPRX2-mediated mast cell degranulation releases numerous mediators, including histamine, tryptase, chymase, serotonin, leukotrienes, prostaglandins, and cytokines such as IL-4, IL-6, and TNF-α, thereby generating a potent inflammatory microenvironment capable of modulating neuronal excitability, vascular permeability, and leukocyte recruitment [96,100,101].

In epithelial tissues (e.g., skin, airway mucosa, and gastrointestinal tract), Mrgprs are expressed in keratinocytes, epithelial progenitors, secretory cells, and some subsets of tissue-resident immune cells [101,102]. Their activation controls epithelial barrier integrity, antimicrobial peptide and cytokine secretion, and wound healing responses [102,103,104,105]. In the gastrointestinal system, expression of Mrgpr family members in enteric neurons, enterochromaffin cells, innate lymphoid cells, and smooth muscle fibers contributes to the regulation of motility, visceral sensitivity, mucosal immune responses, and secretomotor function [89,106,107,108]. Mrgpr-mediated detection of some microbial metabolites and dietary compounds may constitute part of a broader neuroimmune surveillance network that orchestrates host-microbe interactions and drives mucosal homeostasis [109,110]. Dysregulation of these pathways has been implicated in conditions such as irritable bowel syndrome, inflammatory bowel disease, and functional dyspepsia, although mechanistic delineation remains an active area of investigation [82,107,111].

The Mrgpr family is definitively a specialized system integrating neuronal, immune, and epithelial signals to regulate sensory processing and neuroimmune communication. The following section summarizes evidence positioning Mrgprs as key regulators in controlling transcriptional reprogramming, ion-channel modulation, and neuroimmune interactions after nerve injury, driving persistent nociceptor hyperexcitability and maladaptive plasticity.

## 3. Role of Mrgprs in the Pathophysiology of Neuropathic Pain

Members of the Mrgpr family have emerged as important molecular nodes that modulate nociceptor excitability, neuro-immune communication, and maladaptive plasticity following nerve injury. Although originally classified as itch-associated GPCRs, accumulating evidence indicates that numerous Mrgprs participate in pain amplification through finely tuned signaling interactions with several ion channels, neuropeptides, and inflammatory mediators. Neuropathic pain-related dysregulation of Mrgprs reshapes the functional properties of primary sensory neurons and adjacent immune cells, ultimately promoting persistent hyperexcitability and aberrant intercellular communication.

### 3.1. Transcriptional and Epigenetic Remodeling of Mrgprs After Nerve Injury

Peripheral nerve injury initiates a multilayered molecular cascade that produces a deeply remodeled and pathologically permissive Mrgpr signaling landscape across DRG neurons [37,38] and associated immune populations [80,81,82], ultimately driving persistent nociceptor hyperexcitability. Following axotomy, injury-responsive transcription factors are activated in a temporally structured and mechanistically cooperative sequence. ATF3, induced within hours, binds high-affinity stress-responsive elements embedded in Mrgpr promoters and recruits the histone acetyltransferases CBP and p300, facilitating the deposition of activating chromatin marks such as H3K27ac and H3K9ac and thereby enhancing local chromatin accessibility [112,113,114]. Simultaneously, NF-κB is engaged by TNF-α, IL-1β, and related cytokine signals and, after translocating to the nucleus, removes repressive HDAC complexes from Mrgpr regulatory elements while reinforcing promoter-enhancer looping via interactions with architectural proteins, thereby generating a more permissive and functionally arranged transcriptional microenvironment [115]. On the other hand, STAT3, phosphorylated at Tyr705 in response to IL-6 family cytokines, dimerizes and binds canonical STAT response elements within Mrgpr promoters, recruiting several elongation factors that facilitate productive RNA polymerase II progression and sustain high-amplitude transcription [116,117,118,119]. These transcriptional mechanisms are reinforced by ERK1/2- and JNK-dependent activation of c-JUN, EGR1, and other immediate-early genes, which expand the accessible enhancer landscape in both IB4^+^ non-peptidergic nociceptors and TRPV1^+^/CGRP^+^ peptidergic neurons, promoting the ectopic expression of MrgprA3, MrgprC11, MrgprD, MRGPRX1, and MRGPRX3 in some neuronal subsets that normally lack these receptors [44,84,120,121].

### 3.2. Mrgpr-Mediated G Protein Signaling and Ion Channel Modulation

Upon ligand engagement, Mrgprs couple to multiple G protein subtypes, integrating Gαq/11-, Gαi/o-, Gαs-, and Gβγ-dependent pathways into a highly convergent modulatory network that alters nociceptor excitability [46,72,122]. Gαq/11 signaling orchestrates PKC-dependent phosphorylation of some channels (Nav1.7, Nav1.8, Nav1.9, TRPV1, and TRPA1), which shifts activation curves leftward, enhances persistent Na^+^ currents, and reduces the threshold for action-potential initiation [123,124,125,126]. Simultaneously, PIP_2_ depletion destabilizes Nav channel gating and blocks KCNQ2/3-mediated M-currents, removing a major constraint on membrane excitability and promoting spontaneous or high-frequency firing [127,128,129].

Mrgpr signaling further releases Gβγ subunits, which strengthen TRPA1 activation kinetics and cooperate with Gαq pathways to streamline microdomain Ca^2+^ signals near active zones, enabling the spatial control of vesicular release and ion channel phosphorylation [130,131]. In neuropathic conditions, Gαi/o pathways that typically inhibit adenylate cyclase can, through injury-induced feedback, engage AC1 to sustain cAMP production and PKA-dependent channel phosphorylation, exacerbating hyperexcitability [132]. β-arrestin scaffolding of ERK1/2 and p38 MAPK links these acute signaling events to longer-term transcriptional reprogramming, including upregulation of ion channel and neuropeptide genes [133]. In addition, specific receptors (e.g., MrgprD) form direct protein-protein complexes with mechanosensitive Piezo2 channels, amplifying the mechanotransduction process and contributing to mechanical allodynia [134].

### 3.3. Emerging Roles of Mrgprs in Neuroimmune Communication

Peripheral nerve injury engages Mrgprs as crucial organizers of a multilevel neuroimmune circuit that sustains chronic neuropathic hypersensitivity. Peripheral axonal injury induces the recruitment and activation of numerous cell types, including mast cells, macrophages, and Schwann cells, which engage in interconnected reciprocal signaling loops with Mrgpr-expressing nociceptors rather than functioning as isolated effector cells [108].

**Figure 2:**
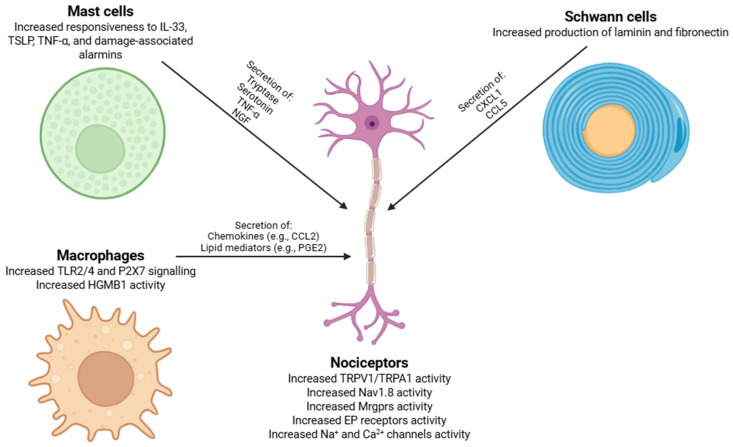
Peripheral nerve injury initiates a bidirectional neuroimmune cascade in which mast cells, macrophages, Schwann cells, and nociceptors engage in tightly coupled signaling loops that drive neuropathic pain. This crosstalk significantly enhances pro-inflammatory mediator release and ion channel remodeling in nociceptors, resulting in persistent hyperexcitability and pathological pain signaling. Abbreviations: IL-33 (interleukin-33), TSLP (thymic stromal lymphopoietin), TNF-α (tumor necrosis factor-alpha), DAMPs (damage-associated molecular patterns), TLR2/4 (Toll-like receptor 2/4), P2X7 (P2X7 purinergic receptor), HMGB1 (high-mobility group box 1), CCL2 (C-C motif chemokine ligand 2), PGE_2_ (prostaglandin E_2_), CXCL1 (C-X-C motif chemokine ligand 1), CCL5 (C-C motif chemokine ligand 5), TRPV1 (transient receptor potential vanilloid 1), TRPA1 (transient receptor potential ankyrin 1), Nav1.8 (voltage-gated sodium channel 1.8), Mrgpr (Mas-related G-protein-coupled receptors), EP receptor (prostaglandin E receptor), Na^+^ (sodium ion), and Ca^2+^ (calcium ion).

Some immune cell types undergo coordinated phenotypic remodeling that strengthens neuro-immune communication, and mast cells (Figure 2) in inflamed tissues show elevated MRGPRX2 expression together with an enhanced responsiveness to diverse peptide mediators (IL-33, TSLP, TNF-α, and damage-associated alarmins), leading via Gαq-dependent Ca^2+^ mobilization to exocytosis of histamine-independent mediators such as tryptase, serotonin, TNF-α, and NGF through Rab27b-, Munc13-4-, and SNARE-dependent fusion machinery [135,136,137]. Tryptase induces ERK and PKCε through PAR2, whereas NGF engages TrkA-PI3K/AKT and ERK signaling pathways [138,139]. Collectively, these events enhance TRPV1/TRPA1 activity, increase Nav1.8 function, and upregulate Mrgpr expression on nociceptors, creating a self-reinforcing excitatory state. Similarly, infiltrating macrophages undergo dynamic reprogramming of receptor and cytokine expression in response to interferon signaling, TLR2/4 activation, P2X7 stimulation, and HMGB1, creating an inflammatory milieu that allows engagement of Mrgpr-dependent pathways [48,89]. Macrophage-derived chemokines (e.g., CCL2) and lipid mediators (e.g., PGE_2_) activate EP receptors on Mrgpr^+^ neurons, eliciting PKC- and PKA-dependent phosphorylation of Na^+^ and Ca^2+^ channels, thereby further enhancing neuronal excitability [140,141].

Finally, following peripheral nerve injury, Schwann cells (Figure 2) undergo a phenotype shift from a myelinating to a repair-promoting state. In this context, Schwann cells upregulate the synthesis and secretion of key extracellular matrix proteins, like laminin and fibronectin [142]. Laminin interacts with integrin receptors on the growth cones of regenerating axons, activating intracellular signaling cascades such as the focal adhesion kinase (FAK) and phosphoinositide 3-kinase (PI3K)/Akt pathways, which allow cytoskeletal reorganization and directed axonal elongation [143]. Fibronectin, through binding to α5β1 integrins, further allows adhesion, migration, and branching of regenerating fibers [144]. Moreover, Schwann cells secrete chemokines, such as CXCL1 and CCL5, which act in both autocrine and paracrine manners to modulate axonal growth and attract macrophages that contribute to debris clearance and growth-promoting cytokine release [145].

These injury-induced signals contribute to the upregulation of several Mrgpr family members on regenerating sensory fibers. This upregulation may involve the activation of transcription factors, including c-Jun and STAT3, which bind to the promoter regions of Mrgpr genes, thereby sensitizing regenerating nociceptive neurons and modulating their responsiveness to inflammatory and pruritogenic stimuli during tissue repair [146,147].

### 3.4. Neuropeptide Release and Peripheral Hyperexcitability

Activation of members of the Mrgpr family in primary afferent nociceptors drives a pronounced Ca^2+^-dependent dysregulation of neurotransmitter and neuropeptide release at peripheral terminals, thereby remodeling the molecular framework of neurogenic inflammation and modulating the synaptic transmission enhancements that underlie neuropathic pain [88,148]. Within this framework, Mrgpr-mediated modulation of presynaptic ion channels and the release machinery synergizes with Nav channel-induced membrane depolarization to augment Ca^2+^ entry, elevating intraterminal Ca^2+^ to supraphysiological concentrations [88,149]. The emergent Ca^2+^ microdomains favor high-affinity synaptotagmin binding, expedite SNARE-complex assembly, and transition synaptic vesicles into a release-ready state, ultimately driving increased exocytosis of substance P, CGRP, galanin, B-type natriuretic peptide (BNP), and other dense-core vesicle cargos far surpassing their typical stimulus-evoked outputs [150,151,152,153]. These neuropeptides, beyond their effects in the dorsal horn, act through their cognate receptors (e.g., NK_1_R, CRLR/RAMP1, and natriuretic peptide receptors) expressed on vascular endothelial cells, perivascular immune cells, and stromal elements of the peripheral inflammatory milieu [154]. Their coordinated activity mediates arteriolar vasodilation, plasma extravasation, endothelial activation, and the directed recruitment of neutrophils, macrophages, and mast cells [155].

Following peripheral nerve injury, Mrgpr^+^ primary afferent nociceptors in laminae I-II of the dorsal horn undergo profound presynaptic and postsynaptic remodeling that potentiates central sensitization [156]. At presynaptic terminals, Cav2.2 (N-type) and Cav3.x (T-type) VGCCs exhibit increased open probability and current density, generating localized Ca^2+^ microdomains that orchestrate synaptotagmin-1/2-mediated vesicle fusion and accelerate SNARE complex assembly (including syntaxin-1, SNAP-25, and VAMP2) at the active zone, with RIM, Munc13, and Bassoon orchestrating vesicle docking and priming to shift the readily releasable pool toward a high-probability release state [157,158,159].

The above-mentioned neurotransmitters couple with postsynaptic NMDA receptors and mGluR5, where activity-dependent phosphorylation by CaMKII, PKC, and Src-family kinases enhances channel open probability, prolongs mean open time, reduces Mg^2+^ block, and increases Ca^2+^ influx, thus driving several downstream signaling cascades [160,161]. Ca^2+^-dependent activation of CaMKII and PKC enables GluA1 Ser831 phosphorylation, promoting AMPA receptor insertion into the postsynaptic density and stabilizing synaptic potentiation [162,163]. Concurrently, ERK1/2 activation and nuclear translocation of phosphorylated CREB enhanced transcription of AMPA (GluA1/2), NMDA (GluN2A/2B) receptor subunits, postsynaptic scaffolds (PSD-95, Homer1b/c, and Shank), and cytoskeletal regulators (cofilin, Rac1, and Arp2/3), resulting in dendritic spine enlargement, increased spine head volume, and stabilization of excitatory synapses [164,165,166].

Finally, excessive glutamatergic and peptidergic input concomitantly diminishes inhibitory synaptic control through retrograde endocannabinoid-mediated suppression of presynaptic release, modulation of GABA/glycine transporter function, and BDNF-driven downregulation of KCC2, thereby generating a state of disinhibition that permits low-threshold mechanoreceptor signals to engage aberrant nociceptive circuits [167,168,169,170,171].

Collectively, Mrgpr activation establishes a feed-forward loop of peripheral and central hyperexcitability that amplifies neurotransmitter and neuropeptide release, remodels synaptic architecture, and promotes maladaptive pain signaling. These insights highlight the therapeutic potential of targeting Mrgprs to disrupt pathological neuropeptide release and hyperexcitability in neuropathic pain.

## 4. Therapeutic Potential of Targeting Mrgprs

Pharmacological strategies to modulate Mrgprs include the use of agonists, antagonists, and biased ligands. While agonists may engage antinociceptive pathways in a subtype-dependent manner, antagonists are currently the most promising approach to inhibit pain-promoting Mrgpr signaling. Table 2 summarizes those pharmacological agents that are currently under investigation in preclinical models and human studies. This table provides an overview of targeted Mrgpr subtypes and their relevance to pain modulation.

Although the contribution of Mrgpr inhibition has not yet been fully characterized in models of neuropathic pain, accumulating evidence indicates that blockade of multiple Mrgpr subtypes produces robust analgesic effects across a variety of inflammatory, visceral, and pruritic pain models. These findings suggest that Mrgprs play a broader role in nociceptive sensitization and peripheral sensory neuron excitability than previously appreciated. As a result, Mrgpr subtypes represent promising molecular targets for the development of novel analgesic therapies, predominantly in conditions where conventional pain treatments are insufficient or associated with significant adverse effects.

Among the pharmacological agents previously cited, aprepitant merits particular attention, given its molecular mechanism, its relevance to neuropeptide-mediated signaling pathways, and its distinctive translational and clinical significance. Aprepitant blocks substance P interaction with neurokinin-1 receptor (NK_1_R), thereby reducing neuronal excitability, mitigating neurogenic inflammation, and suppressing activation of central emetic pathways in the area postrema and nucleus tractus solitarius [177]. Pharmacokinetically, aprepitant is lipophilic, highly protein-bound (>95%), crosses the blood–brain barrier efficiently, and is mainly metabolized by CYP3A4, with additional minor contributions from CYP1A2 and CYP2C19, conferring clinically relevant pharmacokinetic interaction potential [178].

From a receptor pharmacology perspective, aprepitant has been shown in murine systems to inhibit Mrgprb2 [172]; however, its effects on the human ortholog, MRGPRX2, remain undetermined. Clinically, aprepitant has been established as a selective NK_1_R antagonist via phase II-III randomized controlled trials in chemotherapy-induced nausea and vomiting (CINV), where its central NK_1_R blockade synergizes with 5-HT_3_ receptor antagonists and glucocorticoids to increase response rates in both acute and delayed emetic phases [179]. Beyond its antiemetic indication, several exploratory trials have repurposed aprepitant as an antipruritic agent based on the contribution of substance P-NK_1_R signaling to chronic, largely histamine-independent itch. Some studies, like NCT01683552 [180], NCT02646020 [181], NCT03808805 [182], NCT01963793 [183], and NCT01625455 [184], demonstrated efficacy across non-histaminergic pruritic phenotypes.

Conversely, targeting certain Mrgprs (e.g., MRGPRX2) represents a promising strategy for the prevention of pseudo-allergic reactions and mast cell-driven inflammatory pathologies. However, Mrgprs are involved not only in pathological mast cell degranulation but also in protective immunosurveillance, antimicrobial defense, and the maintenance of tissue homeostasis at barrier interfaces [135,185,186]. As a result, pharmacological antagonism of these receptors may be associated with unintended risks, including impairment of immune responses, disruption of host-microbe interactions, and impaired wound-healing processes [135,185,186].

Collectively, these results highlight the therapeutic potential of simultaneously targeting multiple Mrgpr subtypes to elicit potent analgesic and antipruritic effects, particularly in contexts where conventional therapies are limited or associated with side effects.

## 5. Conclusions

Mrgprs have shifted from being a poorly characterized subset of sensory receptors to emerging as crucial regulators of nociceptive pathways and fundamental mediators in the development of neuropathic pain. Increasing evidence supports the notion that specific Mrgpr subtypes play a crucial role in both the onset of neuronal sensitization following nerve injury and the persistence of chronic pain. They exert these effects through diverse mechanisms, including modulation of ion channel function, activation of neuroimmune signaling, and facilitation of mast-cell-neuronal communication that sustains persistent hypersensitivity.

Despite considerable progress, our understanding of Mrgpr biology remains limited. Critical knowledge gaps remain concerning the identity and physiological significance of endogenous and exogenous ligands, the subtype-specific signaling pathways activated in distinct neuronal and non-neuronal cell populations, and the degree to which Mrgpr-mediated signaling intersects with canonical nociceptive mechanisms. Resolving these questions is essential for developing a more comprehensive framework of how Mrgprs modulate sensory processing under both physiological and pathological conditions.

Importantly, the restricted and highly patterned expression of Mrgprs within defined subsets of peripheral sensory neurons positions them as compelling targets for next-generation analgesics. By facilitating therapeutic approaches that target discrete molecular nodes specifically linked to pathological pain, rather than inducing widespread neuronal inhibition, Mrgpr-based interventions have the potential to achieve greater efficacy while reducing systemic side effects. In addition, the predominantly peripheral localization of Mrgprs suggests a therapeutic window for modulating nociceptive signaling without substantial CNS engagement, thereby potentially reducing sedation, cognitive impairment, and abuse liability that characterize centrally acting analgesics, particularly opioids. This peripheral selectivity enables the modulation of pain-associated neuroimmune and sensitization pathways that are not effectively targeted by existing therapies, thereby offering a mechanistically distinct and potentially complementary analgesic strategy. On the other hand, this therapeutic potential is underscored by emerging evidence that pharmacological or genetic modulation of Mrgpr activity can attenuate pain behaviors in several preclinical models of neuropathy. Pharmacological and genetic modulation of Mrgpr activity largely converge in showing decreased nociceptive hypersensitivity, supporting a shared causal role in neuropathic pain. However, partial divergence between these approaches has also been observed, likely due to off-target pharmacological effects, developmental compensatory mechanisms in genetic models, cell-type-specific receptor functions, interspecies differences, and ligand-biased signaling mechanisms.

While Mrgprs represent a promising therapeutic target, it should be acknowledged that the translational development of Mrgpr-targeted interventions remains at an early-stage relative to more established analgesic strategies, such as opioids, CGRP modulators, or anti-NGF monoclonal antibodies. Continued preclinical validation, optimization of ligand specificity, and demonstration of efficacy and safety in clinically relevant models will be essential before Mrgpr-based therapies can reach comparable developmental maturity.

Taken together, current evidence underscores the potential of Mrgprs as both mechanistic biomarkers and therapeutic targets in the context of neuropathic pain. Continued investigation into Mrgpr ligand specificity, intracellular signaling mechanisms, and interactions with the neuroimmune environment will not only enhance our understanding of pain pathophysiology but may also drive the development of more selective and effective therapeutic strategies for a condition that remains exceptionally difficult to manage.

## Figures and Tables

**Table 1:** Distribution of Mrgpr genes across subfamilies in humans and mice.

Subfamily	Human Gene(s)	Mouse Gene(s)
MRGPRA	None	Mrgpra1, Mrgpra2a, Mrgpra2b, Mrgpra3, Mrgpra4, Mrgpra5, Mrgpra6, Mrgpra7, Mrgpra8, Mrgpra9, Mrgpra10
MRGPRB	None	Mrgprb1, Mrgprb2, Mrgprb3, Mrgprb4, Mrgprb5
MRGPRC	None	Mrgprc11
MRGPRD	MRGPRD	Mrgprd
MRGPRE	MRGPRE	Mrgpre
MRGPRF	MRGPRF	Mrgprf
MRGPRG	MRGPRG	Mrgprg
MRGPRX	MRGPRX1, MRGPRX2, MRGPRX3, MRGPRX4	Mrgprx1

**Table 2:** Small-molecule and peptide antagonists targeting human and murine Mrgprs: functional evidence in mast cell degranulation, neurogenic inflammation, and pruritus models. Abbreviations: MRGPRX2 (Mas-related G-protein-coupled receptor X2 -human-), Mrgpra1 (Mas-related G-protein-coupled receptor A1 -mouse-), Mrgprb2 (Mas-related G-protein-coupled receptor B2 -mouse-), Mrgprd (Mas-related G-protein-coupled receptor D -mouse-), QWF (Trp-Gln-Phe tripeptide), TRPA1 (transient receptor potential ankyrin 1), PSB-172656 (3-ethyl-7,8-difluoro-2-isopropylbenzo [4,5]imidazo [1,2-a] pyrimidin-4(1H)-one), Ca^2+^ (calcium ion), and IC_50_ (half maximal inhibitory concentration).

Receptor	Species Tested	Compound	Functional Evidence	References
MRGPRX2	Human	QWF (Trp-Gln-Phe)	QWF inhibits MRGPRX2-mediated degranulation in human mast cells, thereby reducing the release of histamine and proteases that contribute to itch and neurogenic inflammation. Additionally, substance P-induced scratching is effectively blocked by QWF through antagonism of MrgprA1 and MrgprB2 receptors	[172]
Mrgpra1	Mouse			
Mrgprb2				
MRGPRX2	Human	C9 C9-6	C9 and C9-6 are small-molecule MRGPRX2 antagonists that inhibit receptor activation, thus reducing mast cell degranulation in vitro	[173]
		PSB-172656 (3-ethyl-7,8-difluoro-2-isopropylbenzo [4,5]imidazo [1,2-a] pyrimidin-4(1H)-one)	A competitive antagonist that inhibits MRGPRX2-mediated Ca^2+^ mobilization, G protein dissociation, β-arrestin recruitment, and mast cell degranulation	[40]
		Compound A Compound B	Potent antagonists in human mast cells and reporter assays, effectively inhibiting substance P-induced degranulation and associated signaling in vitro and ex vivo	[174]
Mrgprb2	Mouse	C48/80 GE0117 GE0118 GE0119 GE1109 GE1110 GE1111	Library-derived compounds with low micromolar IC_50_ that inhibit MRGPRX2-dependent Ca^2+^ signaling, degranulation, and mediator release, reducing acute allergic responses and systemic anaphylaxis in mice	[175]
MRGPRX2	Human	KMH-45	A non-peptide inhibitor identified through in silico docking and validated by Ca^2+^ flux assays. KMH-45 effectively suppressed MRGPRX2-mast cell activation	[176]
Mrgprb2	Mouse	Aprepitant	Both compounds inhibit substance P-induced MrgprB2 activation and mast cell degranulation, attenuating neurogenic inflammation and itch in murine models	[172]
		L-733,060		
Mrgprd	Mouse	HC-030031	MrgprD plays a significant role in mechanical and cold hypersensitivity in neuropathic pain, with TRPA1-mediated facilitation amplifying MrgprD-dependent nociceptive signaling. On the other hand, the pharmacological inhibition of TRPA1 attenuates MrgprD-associated pain behaviors	[88]

## Data Availability

No new data were generated.

## Abbreviations

The following abbreviations are used in this manuscript:

5-HT_3_	5-hydroxytryptamine type 3 receptor
AC1	Adenylate cyclase 1
AIDS	Acquired immunodeficiency syndrome
AKT	Protein kinase B
ATF3	Activating transcription factor 3
BAM8-22	Bovine adrenal medulla peptide 8-22
BDNF	Brain-derived neurotrophic factor
BNP	B-type natriuretic peptide
Ca^2+^	Calcium ion
Cai^2+^	Intracellular calcium ion concentration
CaMKII	Calcium/calmodulin-dependent protein kinase II
cAMP	Cyclic adenosine monophosphate
Cav2.2	Voltage-gated calcium channel, N-type
Cav3.x	Voltage-gated calcium channel, T-type
CBP	CREB-binding protein
CCL2	C-C motif chemokine ligand 2
CCL3	C-C motif chemokine ligand 3
CCL5	C-C motif chemokine ligand 5
CGRP	Calcitonin gene-related peptide
CINV	Chemotherapy-induced nausea and vomiting
CIPN	Chemotherapy-induced peripheral neuropathy
c-JUN	Jun proto-oncogene, AP-1 transcription factor subunit
CREB	cAMP response element-binding protein
CRLR	Calcitonin receptor-like receptor
CYP3A4	Cytochrome P450 3A4
CYP1A2	Cytochrome P450 1A2
CYP2C19	Cytochrome P450 2C19
CXCL1	C-X-C motif chemokine ligand 1
DAG	Diacylglycerol
DAMP	Damage-associated molecular pattern
DRG	Dorsal root ganglia
DRY motif	Asp-Arg-Tyr motif
EGR1	Early growth response protein 1
EP	Prostaglandin E receptor
ERK	Extracellular signal-regulated kinase
ERK1/2	Extracellular signal-regulated kinase 1/2
FAK	Focal adhesion kinase
GABA	Gamma-aminobutyric acid
GBS	Guillain-Barré Syndrome
GluA1/2	AMPA receptor subunits A1 and A2
GluN2A/2B	NMDA receptor subunits N2A and N2B
GPCR	G-protein-coupled receptor
Gαi/o	G alpha i/o subunit
Gαq	G protein alpha q subunit
Gαq/11	G alpha q/11 subunit
Gαs	G alpha s subunit
Gβγ	G beta-gamma subunit
H3K27ac	Acetylation of lysine 27 on histone H3
H3K9ac	Acetylation of lysine 9 on histone H3
HDAC	Histone deacetylase
HIV	Human immunodeficiency virus
HMGB1	High-mobility group box 1
IASP	International Association for the Study of Pain
IB4	Isolectin B4
IC_50_	Half maximal inhibitory concentration
IgE	Immunoglobulin E
IL-1β	Interleukin 1 beta
IL-33	Interleukin 33
IL-4	Interleukin 4
IL-6	Interleukin 6
IP_3_	Inositol 1,4,5-trisphosphate
IP_3_R	IP_3_ receptor
JNK	c-Jun N-terminal kinase
K^+^	Potassium ion
KCC2	Potassium-chloride cotransporter 2
KCNQ2/3	M-type potassium channel subunits 2 and 3
LL-37	Cathelicidin antimicrobial peptide LL-37
MAPK	Mitogen-activated protein kinase
mGluR5	Metabotropic glutamate receptor 5
Mrgpr	Mas-related G-protein-coupled receptor
Mrgpra1	Mas-related G-protein-coupled receptor, subtype A1 (mouse)
Mrgpra10	Mas-related G-protein-coupled receptor, subtype A10 (mouse)
Mrgpra2a	Mas-related G-protein-coupled receptor, subtype A2a (mouse)
Mrgpra2b	Mas-related G-protein-coupled receptor, subtype A2b (mouse)
Mrgpra3	Mas-related G-protein-coupled receptor, subtype A3 (mouse)
Mrgpra4	Mas-related G-protein-coupled receptor, subtype A4 (mouse)
Mrgpra5	Mas-related G-protein-coupled receptor, subtype A5 (mouse)
Mrgpra6	Mas-related G-protein-coupled receptor, subtype A6 (mouse)
Mrgpra7	Mas-related G-protein-coupled receptor, subtype A7 (mouse)
Mrgpra8	Mas-related G-protein-coupled receptor, subtype A8 (mouse)
Mrgpra9	Mas-related G-protein-coupled receptor, subtype A9 (mouse)
Mrgprb1	Mas-related G-protein-coupled receptor, subtype B1 (mouse)
Mrgprb2	Mas-related G-protein-coupled receptor, subtype B2 (mouse)
Mrgprb3	Mas-related G-protein-coupled receptor, subtype B3 (mouse)
Mrgprb4	Mas-related G-protein-coupled receptor, subtype B4 (mouse)
Mrgprb5	Mas-related G-protein-coupled receptor, subtype B5 (mouse)
Mrgprc11	Mas-related G-protein-coupled receptor, subtype C11 (mouse)
MRGPRD	Mas-related G-protein-coupled receptor D (human)
Mrgprd	Mas-related G-protein-coupled receptor D (mouse)
MRGPRE	Mas-related G-protein-coupled receptor E (human)
Mrgpre	Mas-related G-protein-coupled receptor E (mouse)
MRGPRF	Mas-related G-protein-coupled receptor F (human)
Mrgprf	Mas-related G-protein-coupled receptor F (mouse)
MRGPRG	Mas-related G-protein-coupled receptor G (human)
Mrgprg	Mas-related G-protein-coupled receptor G (mouse)
MRGPRX1	Mas-related G-protein-coupled receptor X1 (human)
Mrgprx1	Mas-related G-protein-coupled receptor X1 (mouse)
MRGPRX2	Mas-related G-protein-coupled receptor X2 (human)
MRGPRX3	Mas-related G-protein-coupled receptor X3 (human)
MRGPRX4	Mas-related G-protein-coupled receptor X4 (human)
Munc13-4	Mammalian uncoordinated-13-4
Nav	Voltage-gated sodium channel
Nav1.7	Voltage-gated sodium channel 1.7
Nav1.8	Voltage-gated sodium channel 1.8
Nav1.9	Voltage-gated sodium channel 1.9
NF-κB	Nuclear factor kappa-light-chain-enhancer of activated B cells
NGF	Nerve growth factor
NK1	Neurokinin 1 receptor
NMDA	N-methyl-D-aspartate
NPxxY motif	Asp-Pro-x-x-Tyr motif
NR2B	NMDA receptor subunit 2B
P2 × 7	Adenosine triphosphate receptor P2 × 7
p300	Histone acetyltransferase p300
p38 MAPK	p38 mitogen-activated protein kinase
PAR2	Protease-activated receptor 2
PGE_2_	Prostaglandin E2
PI3K	Phosphoinositide 3-kinase
PIP_2_	Phosphatidylinositol 4,5-bisphosphate
PKA	Protein kinase A
PKC	Protein kinase C
PKCε	Protein kinase C epsilon
PLC	Phospholipase C
PLCβ	Phospholipase C beta
PSB-172656	3-ethyl-7,8-difluoro-2-isopropylbenzo [4,5]imidazo [1,2-a] pyrimidin-4(1H)-one
PSD-95	Postsynaptic density protein 95
QWF	Trp-Gln-Phe tripeptide
Rab27b	Ras-related protein Rab-27B
RAMP1	Receptor activity–modifying protein 1
RIM	Rab3-interacting molecule
SLIGR	Ser-Leu-Ile-Gly-Arg-Leu peptide
SNARE	Soluble NSF (N-ethylmaleimide–sensitive factor) attachment protein receptor
SNRI	Serotonin-noradrenaline reuptake inhibitor
STAT3	Signal transducer and activator of transcription 3
TCA	Tricyclic antidepressant
TLR2	Toll-like receptor 2
TLR4	Toll-like receptor 4
TM3	Transmembrane domain 3
TM7	Transmembrane domain 7
TNF-α	Tumor necrosis factor alpha
TrkA	Tropomyosin receptor kinase A
TRP	Transient receptor potential
TRPA1	Transient receptor potential ankyrin 1
TRPV1	Transient receptor potential vanilloid 1
TSLP	Thymic stromal lymphopoietin
VAMP2	Vesicle-associated membrane protein 2
VGCC	Voltage-gated calcium channel
VGSC	Voltage-gated sodium channel
α5β1	Integrin alpha 5 beta 1

## References

[1] Raja S.N., Carr D.B., Cohen M., Finnerup N.B., Flor H., Gibson S., Keefe F.J., Mogil J.S., Ringkamp M., Sluka K.A. (2020). The revised International Association for the Study of Pain definition of pain: Concepts, challenges, and compromises. Pain.

[2] Vader K., Bostick G.P., Carlesso L.C., Hunter J., Mesaroli G., Perreault K., Tousignant-Laflamme Y., Tupper S., Walton D.M., Wideman T.H. (2021). The Revised IASP Definition of Pain and Accompanying Notes: Considerations for the Physiotherapy Profession. Physiother. Can..

[3] Fashler S.R., Cooper L.K., Oosenbrug E.D., Burns L.C., Razavi S., Goldberg L., Katz J. (2016). Systematic Review of Multidisciplinary Chronic Pain Treatment Facilities. Pain Res. Manag..

[4] Forte G., Favieri F., De Pascalis V., Casagrande M. (2024). To Be in Pain: Pain Multidimensional Questionnaire as Reliable Tool to Evaluate Multifaceted Aspects of Pain. J. Clin. Med..

[5] Sima S., Lapkin S., Gan Z., Diwan A.D. (2024). Nociceptive pain assessed by the PainDETECT questionnaire may predict response to opioid treatment for chronic low back pain. Heliyon.

[6] Kaplan C.M., Kelleher E., Irani A., Schrepf A., Clauw D.J., Harte S.E. (2024). Deciphering nociplastic pain: Clinical features, risk factors and potential mechanisms. Nat. Rev. Neurol..

[7] van Hecke O., Austin S.K., Khan R.A., Smith B.H., Torrance N. (2014). Neuropathic pain in the general population: A systematic review of epidemiological studies. Pain.

[8] Martins J.P., Marson F.A.L. (2024). A narrative review of the complex panorama regarding chronic neuropathic pain mainly for the psychological issues. Heliyon.

[9] Madariaga Muñoz M.C., Villegas Estévez F., Jiménez López A.J., Cabezón Álvarez A., Soler López B. (2018). Evaluation of Quality of Life and Satisfaction of Patients with Neuropathic Pain and Breakthrough Pain: Economic Impact Based on Quality of Life. Pain Res. Treat..

[10] Miclescu A., Rönngren C., Bengtsson M., Gordh T., Hedin A. (2024). Increased risk of persistent neuropathic pain after traumatic nerve injury and surgery for carriers of a human leukocyte antigen haplotype. Pain.

[11] Mattar M., Umutoni F., Hassan M.A., Wamburu M.W., Turner R., Patton J.S., Chen X., Lei W. (2024). Chemotherapy-Induced Peripheral Neuropathy: A Recent Update on Pathophysiology and Treatment. Life.

[12] Zhu J., Hu Z., Luo Y., Liu Y., Luo W., Du X., Luo Z., Hu J., Peng S. (2024). Diabetic peripheral neuropathy: Pathogenetic mechanisms and treatment. Front. Endocrinol..

[13] Xu S., Li H., Ai Z., Guo R., Cheng H., Wang Y. (2024). Exploring viral neuropathic pain: Molecular mechanisms and therapeutic implications. PLoS Pathog..

[14] Caballero-Ávila M., Pascual-Goñi E., Lleixà C., Martín-Aguilar L., Collet-Vidiella R., Querol L. (2025). The changing landscape of primary autoimmune neuropathies. Nat. Rev. Neurol..

[15] Li J., Kang W., Wang X., Pan F. (2024). Progress in treatment of pathological neuropathic pain after spinal cord injury. Front. Neurol..

[16] Fu Y., Gong C., Zhu C., Zhong W., Guo J., Chen B. (2023). Research trends and hotspots of neuropathic pain in neurodegenerative diseases: A bibliometric analysis. Front. Immunol..

[17] Leone C.M., Truini A. (2024). Understanding neuropathic pain: The role of neurophysiological tests in unveiling underlying mechanisms. J. Anesth. Analg. Crit. Care.

[18] Zhang S., Ning Y., Yang Y., Mu G., Yang Y., Ren C., Liao C., Ou C., Zhang Y. (2025). Decoding pain chronification: Mechanisms of the acute-to-chronic transition. Front. Mol. Neurosci..

[19] Jayathilake N.J., Phan T.T., Kim J., Lee K.P., Park J.M. (2025). Modulating neuroplasticity for chronic pain relief: Noninvasive neuromodulation as a promising approach. Exp. Mol. Med..

[20] Bak M.S., Park H., Kim S.K. (2021). Neural Plasticity in the Brain during Neuropathic Pain. Biomedicines.

[21] Araldi D., Khomula E.V., Bonet I.J.M., Bogen O., Green P.G., Levine J.D. (2024). Role of pattern recognition receptors in chemotherapy-induced neuropathic pain. Brain.

[22] Prado J., Westerink R.H.S., Popov-Celeketic J., Steen-Louws C., Pandit A., Versteeg S., van de Worp W., Kanters D.H.A.J., Reedquist K.A., Koenderman L. (2021). Cytokine receptor clustering in sensory neurons with an engineered cytokine fusion protein triggers unique pain resolution pathways. Proc. Natl. Acad. Sci. USA.

[23] White F.A., Feldman P., Miller R.J. (2009). Chemokine signaling and the management of neuropathic pain. Mol. Interv..

[24] Donnelly C.R., Andriessen A.S., Chen G., Wang K., Jiang C., Maixner W., Ji R.R. (2020). Central Nervous System Targets: Glial Cell Mechanisms in Chronic Pain. Neurotherapeutics.

[25] García-Domínguez M. (2025). Interplay Between Aging and Glial Cell Dysfunction: Implications for CNS Health. Life.

[26] Bennett D.L., Clark A.J., Huang J., Waxman S.G., Dib-Hajj S.D. (2019). The Role of Voltage-Gated Sodium Channels in Pain Signaling. Physiol. Rev..

[27] Alles S.R.A., Smith P.A. (2021). Peripheral Voltage-Gated Cation Channels in Neuropathic Pain and Their Potential as Therapeutic Targets. Front. Pain Res..

[28] Busserolles J., Tsantoulas C., Eschalier A., López García J.A. (2016). Potassium channels in neuropathic pain: Advances, challenges, and emerging ideas. Pain.

[29] Kaye A.D., Armistead G., Amedio L.S., Manthei M.E., Ahmadzadeh S., Bernhardt B., Shekoohi S. (2025). Evolving Treatment Strategies for Neuropathic Pain: A Narrative Review. Medicina.

[30] Soliman N., Moisset X., Ferraro M.C., de Andrade D.C., Baron R., Belton J., Bennett D.L.H., Calvo M., Dougherty P., Gilron I. (2025). Pharmacotherapy and non-invasive neuromodulation for neuropathic pain: A systematic review and meta-analysis. Lancet Neurol..

[31] McNicol E.D., Midbari A., Eisenberg E. (2013). Opioids for neuropathic pain. Cochrane Database Syst. Rev..

[32] Sánchez-Robles E.M., Girón R., Paniagua N., Rodríguez-Rivera C., Pascual D., Goicoechea C. (2021). Monoclonal Antibodies for Chronic Pain Treatment: Present and Future. Int. J. Mol. Sci..

[33] da Cunha P.H.M., Lapa J.D.D.S., Hosomi K., de Andrade D.C. (2024). Neuromodulation for neuropathic pain. Int. Rev. Neurobiol..

[34] Yang D., Zhou Q., Labroska V., Qin S., Darbalaei S., Wu Y., Yuliantie E., Xie L., Tao H., Cheng J. (2021). G protein-coupled receptors: Structure- and function-based drug discovery. Signal. Transduct. Target Ther..

[35] Liu S., Anderson P.J., Rajagopal S., Lefkowitz R.J., Rockman H.A. (2024). G Protein-Coupled Receptors: A Century of Research and Discovery. Circ Res..

[36] Sanchez-Reyes O.B., Zilberg G., McCorvy J.D., Wacker D. (2023). Molecular insights into GPCR mechanisms for drugs of abuse. J. Biol. Chem..

[37] Guan Y., Liu Q., Tang Z., Raja S.N., Anderson D.J., Dong X. (2010). Mas-related G-protein-coupled receptors inhibit pathological pain in mice. Proc. Natl. Acad. Sci. USA.

[38] George D.S., Jayaraj N.D., Pacifico P., Ren D., Sriram N., Miller R.E., Malfait A.M., Miller R.J., Menichella D.M. (2024). The Mas-related G protein-coupled receptor d (Mrgprd) mediates pain hypersensitivity in painful diabetic neuropathy. Pain.

[39] Bader M., Alenina N., Andrade-Navarro M.A., Santos R.A. (2014). MAS and its related G protein-coupled receptors, Mrgprs. Pharmacol. Rev..

[40] Al Hamwi G., Alnouri M.W., Verdonck S., Leonczak P., Chaki S., Frischbutter S., Kolkhir P., Matthey M., Kopp C., Bednarski M. (2025). Subnanomolar MAS-related G protein-coupled receptor-X2/B2 antagonists with efficacy in human mast cells and disease models. Signal. Transduct. Target Ther..

[41] Zhu Y., Hanson C.E., Liu Q., Han L. (2017). Mrgprs activation is required for chronic itch conditions in mice. Itch.

[42] Castro J., Harrington A.M., Lieu T., Garcia-Caraballo S., Maddern J., Schober G., O’Donnell T., Grundy L., Lumsden A.L., Miller P. (2019). Activation of pruritogenic TGR5, MrgprA3, and MrgprC11 on colon-innervating afferents induces visceral hypersensitivity. JCI Insight..

[43] Tseng P.Y., Zheng Q., Li Z., Dong X. (2019). MrgprX1 Mediates Neuronal Excitability and Itch Through Tetrodotoxin-Resistant Sodium Channels. Itch.

[44] Gan B., Yu L., Yang H., Jiao H., Pang B., Chen Y., Wang C., Lv R., Hu H., Cao Z. (2023). Mechanism of agonist-induced activation of the human itch receptor MRGPRX1. PLoS Biol..

[45] Guo L., Zhang Y., Fang G., Tie L., Zhuang Y., Xue C., Liu Q., Zhang M., Zhu K., You C. (2023). Ligand recognition and G protein coupling of the human itch receptor MRGPRX1. Nat. Commun..

[46] Cao C., Roth B.L. (2023). The structure, function, and pharmacology of MRGPRs. Trends Pharmacol. Sci..

[47] Grundy L., Caldwell A., Garcia-Caraballo S., Grundy D., Spencer N.J., Dong X., Castro J., Harrington A.M., Brierley S.M. (2021). Activation of MrgprA3 and MrgprC11 on Bladder-Innervating Afferents Induces Peripheral and Central Hypersensitivity to Bladder Distension. J. Neurosci..

[48] Meixiong J., Dong X. (2017). Mas-Related G Protein-Coupled Receptors and the Biology of Itch Sensation. Annu. Rev. Genet..

[49] Meixiong J., Anderson M., Limjunyawong N., Sabbagh M.F., Hu E., Mack M.R., Oetjen L.K., Wang F., Kim B.S., Dong X. (2019). Activation of Mast-Cell-Expressed Mas-Related G-Protein-Coupled Receptors Drives Non-histaminergic Itch. Immunity.

[50] Green D.P. (2021). The role of Mrgprs in pain. Neurosci. Lett..

[51] Ji Z., Li J., Tao S., Li H., Kong X., Huang B., Feng Z., Wei X., Zheng Z., Chen J. (2024). Mrgprb2-mediated mast cell activation exacerbates Modic changes by regulating immune niches. Exp. Mol. Med..

[52] Dong F.L., Yu L., Feng P.D., Ren J.X., Bai X.H., Lin J.Q., Cao D.L., Deng Y.T., Zhang Y., Shen H.H. (2025). An atlas of neuropathic pain-associated molecular pathological characteristics in the mouse spinal cord. Commun. Biol..

[53] Jiang Y., Ye F., Zhang J., Huang Y., Zong Y., Chen F., Yang Y., Zhu C., Yang T., Yu G. (2025). Dual function of MrgprB2 receptor-dependent neural immune axis in chronic pain. J. Adv. Res..

[54] Uniyal A., Tiwari V., Tsukamoto T., Dong X., Guan Y., Raja S.N. (2023). Targeting sensory neuron GPCRs for peripheral neuropathic pain. Trends Pharmacol. Sci..

[55] Gour N., Dong X. (2024). The MRGPR family of receptors in immunity. Immunity.

[56] Zylka M.J., Dong X., Southwell A.L., Anderson D.J. (2003). Atypical expansion in mice of the sensory neuron-specific Mrg G protein-coupled receptor family. Proc. Natl. Acad. Sci. USA.

[57] Flegel C., Schöbel N., Altmüller J., Becker C., Tannapfel A., Hatt H., Gisselmann G. (2015). RNA-Seq Analysis of Human Trigeminal and Dorsal Root Ganglia with a Focus on Chemoreceptors. PLoS ONE.

[58] Ray P., Torck A., Quigley L., Wangzhou A., Neiman M., Rao C., Lam T., Kim J.Y., Kim T.H., Zhang M.Q. (2018). Comparative transcriptome profiling of the human and mouse dorsal root ganglia: An RNA-seq-based resource for pain and sensory neuroscience research. Pain.

[59] Li Z., Tseng P.Y., Tiwari V., Xu Q., He S.Q., Wang Y., Zheng Q., Han L., Wu Z., Blobaum A.L. (2017). Targeting human Mas-related G protein-coupled receptor X1 to inhibit persistent pain. Proc. Natl. Acad. Sci. USA.

[60] Wang K., Wang S., Chen Y., Wu D., Hu X., Lu Y., Wang L., Bao L., Li C., Zhang X. (2021). Single-cell transcriptomic analysis of somatosensory neurons uncovers temporal development of neuropathic pain. Cell Res..

[61] Wang C., Liu Y., Lanier M., Yeager A., Singh I., Gumpper R.H., Krumm B.E., DeLeon C., Zhang S., Boehm M. (2024). High-affinity agonists reveal recognition motifs for the MRGPRD GPCR. Cell Rep..

[62] Suzuki S., Iida M., Hiroaki Y., Tanaka K., Kawamoto A., Kato T., Oshima A. (2022). Structural insight into the activation mechanism of MrgD with heterotrimeric Gi-protein revealed by cryo-EM. Commun. Biol..

[63] Corbière A., Loste A., Gaudenzio N. (2021). MRGPRX2 sensing of cationic compounds-A bridge between nociception and skin diseases?. Exp. Dermatol..

[64] Subramanian H., Gupta K., Ali H. (2016). Roles of Mas-related G protein-coupled receptor X2 on mast cell-mediated host defense, pseudoallergic drug reactions, and chronic inflammatory diseases. J. Allergy Clin. Immunol..

[65] Reddy V.B., Sun S., Azimi E., Elmariah S.B., Dong X., Lerner E.A. (2015). Redefining the concept of protease-activated receptors: Cathepsin S evokes itch via activation of Mrgprs. Nat. Commun..

[66] Yu H., Zhao T., Liu S., Wu Q., Johnson O., Wu Z., Zhuang Z., Shi Y., Peng L., He R. (2019). MRGPRX4 is a bile acid receptor for human cholestatic itch. elife.

[67] Elst J., Maurer M., Sabato V., Faber M.A., Bridts C.H., Mertens C., Van Houdt M., Van Gasse A.L., van der Poorten M.M., De Puysseleyr L.P. (2021). Novel Insights on MRGPRX2-Mediated Hypersensitivity to Neuromuscular Blocking Agents And Fluoroquinolones. Front. Immunol..

[68] Yang F., Limjunyawong N., Peng Q., Schroeder J.T., Saini S., MacGlashan D., Dong X., Gao L. (2022). Biological screening of a unique drug library targeting MRGPRX2. Front. Immunol..

[69] Reddy V.B., Lerner E.A. (2017). Activation of mas-related G-protein-coupled receptors by the house dust mite cysteine protease Der p1 provides a new mechanism linking allergy and inflammation. J. Biol. Chem..

[70] Al Hamwi G., Riedel Y.K., Clemens S., Namasivayam V., Thimm D., Müller C.E. (2022). MAS-related G protein-coupled receptors X (MRGPRX): Orphan GPCRs with potential as targets for future drugs. Pharmacol. Ther..

[71] Solinski H.J., Zierler S., Gudermann T., Breit A. (2012). Human sensory neuron-specific Mas-related G protein-coupled receptors-X1 sensitize and directly activate transient receptor potential cation channel V1 via distinct signaling pathways. J. Biol. Chem..

[72] Steele H.R., Han L. (2021). The signaling pathway and polymorphisms of Mrgprs. Neurosci. Lett..

[73] Woll K.A., Van Petegem F. (2022). Calcium-release channels: Structure and function of IP_3_ receptors and ryanodine receptors. Physiol. Rev..

[74] Wang Z., Li Z., Bal G., Franke K., Zuberbier T., Babina M. (2022). β-arrestin-1 and β-arrestin-2 Restrain MRGPRX2-Triggered Degranulation and ERK1/2 Activation in Human Skin Mast Cells. Front. Allergy.

[75] Jean-Charles P.Y., Kaur S., Shenoy S.K. (2017). G Protein-Coupled Receptor Signaling Through β-Arrestin-Dependent Mechanisms. J. Cardiovasc. Pharmacol..

[76] Roy S., Gupta K., Ganguly A., Ali H. (2019). β-Arrestin2 expressed in mast cells regulates ciprofloxacin-induced pseudoallergy and IgE-mediated anaphylaxis. J. Allergy Clin. Immunol..

[77] Babina M., Wang Z., Roy S., Guhl S., Franke K., Artuc M., Ali H., Zuberbier T. (2021). MRGPRX2 Is the Codeine Receptor of Human Skin Mast Cells: Desensitization through β-Arrestin and Lack of Correlation with the FcεRI Pathway. J. Investig. Dermatol..

[78] Camunas-Soler J. (2023). Integrating single-cell transcriptomics with cellular phenotypes: Cell morphology, Ca^2+^ imaging and electrophysiology. Biophys. Rev..

[79] Kupari J., Usoskin D., Parisien M., Lou D., Hu Y., Fatt M., Lönnerberg P., Spångberg M., Eriksson B., Barkas N. (2021). Single cell transcriptomics of primate sensory neurons identifies cell types associated with chronic pain. Nat. Commun..

[80] Serhan N., Cenac N., Basso L., Gaudenzio N. (2021). Mas-related G protein-coupled receptors (Mrgprs)—Key regulators of neuroimmune interactions. Neurosci. Lett..

[81] Inclan-Rico J.M., Napuri C.M., Lin C., Hung L.Y., Ferguson A.A., Liu X., Wu Q., Pastore C.F., Stephenson A., Femoe U.M. (2024). MrgprA3 neurons drive cutaneous immunity against helminths through selective control of myeloid-derived IL-33. Nat. Immunol..

[82] Sun N., Cao L.S., Xia W.Y., Wang J.M., Wu Q.F. (2025). Gut sensory neurons as regulators of neuro-immune-microbial interactions: From molecular mechanisms to precision therapy for IBD/IBS. J. Neuroinflamm..

[83] Han L., Ma C., Liu Q., Weng H.J., Cui Y., Tang Z., Kim Y., Nie H., Qu L., Patel K.N. (2013). A subpopulation of nociceptors specifically linked to itch. Nat. Neurosci..

[84] Xing Y., Chen J., Hilley H., Steele H., Yang J., Han L. (2020). Molecular Signature of Pruriceptive MrgprA3^+^ Neurons. J. Investig. Dermatol..

[85] Llimós-Aubach J., Andres-Bilbe A., Pujol-Coma A., Castellanos A., Pallás I., Bahamonde M.I., de Anta J.M., Soler C., Comes N., Callejo G. (2025). TRESK background potassium channel regulates MrgprA3 + pruriceptor excitability, acute and chronic itch. Pain.

[86] Sanjel B., Maeng H.J., Shim W.S. (2019). BAM8-22 and its receptor MRGPRX1 may attribute to cholestatic pruritus. Sci. Rep..

[87] Sikand P., Dong X., LaMotte R.H. (2011). BAM8-22 peptide produces itch and nociceptive sensations in humans independent of histamine release. J. Neurosci..

[88] Wang C., Gu L., Ruan Y., Geng X., Xu M., Yang N., Yu L., Jiang Y., Zhu C., Yang Y. (2019). Facilitation of MrgprD by TRP-A1 promotes neuropathic pain. FASEB J..

[89] Zhou C., Li J., Liu L., Tang Z., Wan F., Lan L. (2019). Expression and localization of MrgprD in mouse intestinal tract. Cell Tissue Res..

[90] Steele H.R., Xing Y., Zhu Y., Hilley H.B., Lawson K., Nho Y., Niehoff T., Han L. (2021). MrgprC11^+^ sensory neurons mediate glabrous skin itch. Proc. Natl. Acad. Sci. USA.

[91] Fujii K., Miyagawa R., Tanaka R., Saito M., Tanaka S., Shiratori-Hayashi M., Tsuda M., Dong X., Fujii M. (2024). MrgprA3^+^ Primary Sensory Neurons Mediate Acute Allergic Itch Responses in Atopic Dermatitis Model Mice. Biol. Pharm. Bull..

[92] Ruppenstein A., Limberg M.M., Loser K., Kremer A.E., Homey B., Raap U. (2021). Involvement of Neuro-Immune Interactions in Pruritus With Special Focus on Receptor Expressions. Front. Med..

[93] Wang Z., Babina M. (2020). MRGPRX2 signals its importance in cutaneous mast cell biology: Does MRGPRX2 connect mast cells and atopic dermatitis?. Exp. Dermatol..

[94] Che D., Zheng Y., Hou Y., Du X., Jia T., Zhao Q., Song X., Zhou T., Geng S. (2021). Action of substance P and PAMP(9-20) on different excitation sites of MRGPRX2 induces differences in mast cell activation. Int. Immunopharmacol..

[95] Zhang F., Hong F., Wang L., Fu R., Qi J., Yu B. (2022). MrgprX2 regulates mast cell degranulation through PI3K/AKT and PLCγ signaling in pseudo-allergic reactions. Int. Immunopharmacol..

[96] Roy S., Chompunud Na Ayudhya C., Thapaliya M., Deepak V., Ali H. (2021). Multifaceted MRGPRX2: New insight into the role of mast cells in health and disease. J. Allergy Clin. Immunol..

[97] Raj S., Lu L., Unsworth L.D. (2021). Screening Peptides that Activate MRGPRX2 using Engineered HEK Cells. J. Vis. Exp..

[98] Porebski G., Kwiecien K., Pawica M., Kwitniewski M. (2018). Mas-Related G Protein-Coupled Receptor-X2 (MRGPRX2) in Drug Hypersensitivity Reactions. Front. Immunol..

[99] Lafleur M.A., Werner J., Fort M., Lobenhofer E.K., Balazs M., Goyos A. (2020). MRGPRX2 activation as a rapid, high-throughput mechanistic-based approach for detecting peptide-mediated human mast cell degranulation liabilities. J. Immunotoxicol..

[100] West P.W., Chéret J., Bahri R., Kiss O., Wu Z., Macphee C.H., Bulfone-Paus S. (2024). The MRGPRX2-substance P pathway regulates mast cell migration. iScience.

[101] Suzuki Y., Shuang L., Takemasa E., Takasaki Y., Yorozuya T., Mogi M. (2025). MRGPRX2-Mediated Mast Cell Degranulation by Monomethyl Methacrylate: Unveiling a Pathway in Bone Cement Implantation Syndrome. Clin. Exp. Pharmacol. Physiol..

[102] Tseng P.Y., Hoon M.A. (2022). GPR15L is an epithelial inflammation-derived pruritogen. Sci. Adv..

[103] Dong X., Limjunyawong N., Sypek E.I., Wang G., Ortines R.V., Youn C., Alphonse M.P., Dikeman D., Wang Y., Lay M. (2022). Keratinocyte-derived defensins activate neutrophil-specific receptors Mrgpra2a/b to prevent skin dysbiosis and bacterial infection. Immunity.

[104] Wong T.K., Choi Y.G., Li P.H., Chow B.K.C., Kumar M. (2024). MRGPRX2 antagonist GE1111 attenuated DNFB-induced atopic dermatitis in mice by reducing inflammatory cytokines and restoring skin integrity. Front. Immunol..

[105] Plum T., Feyerabend T.B., Rodewald H.R. (2024). Beyond classical immunity: Mast cells as signal converters between tissues and neurons. Immunity.

[106] Inclan-Rico J.M., Kim B.S., Abdus-Saboor I. (2021). Beyond somatosensation: Mrgprs in mucosal tissues. Neurosci. Lett..

[107] Hawker P., Zhang L., Liu L. (2024). Mas-related G protein-coupled receptors in gastrointestinal dysfunction and inflammatory bowel disease: A review. Br. J. Pharmacol..

[108] Van Remoortel S., Lambeets L., Timmermans J.P. (2023). Neuro-immune interactions and the role of Mas-related G protein-coupled receptors in the gastrointestinal tract. Anat. Rec..

[109] Uhlig F., Grundy L., Garcia-Caraballo S., Brierley S.M., Foster S.J., Grundy D. (2020). Identification of a Quorum Sensing-Dependent Communication Pathway Mediating Bacteria-Gut-Brain Cross Talk. iScience.

[110] Chang P.V. (2024). Microbial metabolite-receptor interactions in the gut microbiome. Curr. Opin. Chem. Biol..

[111] Padhi P., Worth C., Zenitsky G., Jin H., Sambamurti K., Anantharam V., Kanthasamy A., Kanthasamy A.G. (2022). Mechanistic Insights Into Gut Microbiome Dysbiosis-Mediated Neuroimmune Dysregulation and Protein Misfolding and Clearance in the Pathogenesis of Chronic Neurodegenerative Disorders. Front. Neurosci..

[112] Hunt D., Raivich G., Anderson P.N. (2012). Activating transcription factor 3 and the nervous system. Front. Mol. Neurosci..

[113] Zhao J., Li X., Guo M., Yu J., Yan C. (2016). The common stress responsive transcription factor ATF3 binds genomic sites enriched with p300 and H3K27ac for transcriptional regulation. BMC Genom..

[114] Renthal W., Tochitsky I., Yang L., Cheng Y.C., Li E., Kawaguchi R., Geschwind D.H., Woolf C.J. (2020). Transcriptional Reprogramming of Distinct Peripheral Sensory Neuron Subtypes after Axonal Injury. Neuron.

[115] Arora R., Van Theemsche K.M., Van Remoortel S., Snyders D.J., Labro A.J., Timmermans J.P. (2021). Constitutive, Basal, and β-Alanine-Mediated Activation of the Human Mas-Related G Protein-Coupled Receptor D Induces Release of the Inflammatory Cytokine IL-6 and Is Dependent on NF-κB Signaling. Int. J. Mol. Sci..

[116] Dziennis S., Alkayed N.J. (2008). Role of signal transducer and activator of transcription 3 in neuronal survival and regeneration. Rev. Neurosci..

[117] Leibinger M., Andreadaki A., Diekmann H., Fischer D. (2013). Neuronal STAT3 activation is essential for CNTF- and inflammatory stimulation-induced CNS axon regeneration. Cell Death Dis..

[118] Takahashi S., Ochiai S., Jin J., Takahashi N., Toshima S., Ishigame H., Kabashima K., Kubo M., Nakayama M., Shiroguchi K. (2023). Sensory neuronal STAT3 is critical for IL-31 receptor expression and inflammatory itch. Cell Rep..

[119] Fujinaga K., Huang F., Peterlin B.M. (2023). P-TEFb: The master regulator of transcription elongation. Mol. Cell..

[120] Zhao Z.Q., Huo F.Q., Jeffry J., Hampton L., Demehri S., Kim S., Liu X.Y., Barry D.M., Wan L., Liu Z.C. (2013). Chronic itch development in sensory neurons requires BRAF signaling pathways. J. Clin. Investig..

[121] Yin L., Xu X., Conacher B., Lin Y., Carrillo G.L., Cun Y., Fox M.A., Lu X., Xie H. (2025). Elevated EGR1 binding at enhancers in excitatory neurons correlates with neuronal subtype-specific epigenetic regulation. BMC Biol..

[122] Rau K.K., McIlwrath S.L., Wang H., Lawson J.J., Jankowski M.P., Zylka M.J., Anderson D.J., Koerber H.R. (2009). Mrgprd enhances excitability in specific populations of cutaneous murine polymodal nociceptors. J. Neurosci..

[123] Vijayaragavan K., Boutjdir M., Chahine M. (2004). Modulation of Nav1.7 and Nav1.8 peripheral nerve sodium channels by protein kinase A and protein kinase C. J. Neurophysiol..

[124] Wu D.F., Chandra D., McMahon T., Wang D., Dadgar J., Kharazia V.N., Liang Y.J., Waxman S.G., Dib-Hajj S.D., Messing R.O. (2012). PKCε phosphorylation of the sodium channel NaV1.8 increases channel function and produces mechanical hyperalgesia in mice. J. Clin. Investig..

[125] Liu L., Yudin Y., Rohacs T. (2020). Diacylglycerol kinases regulate TRPV1 channel activity. J. Biol. Chem..

[126] Hall B.E., Prochazkova M., Sapio M.R., Minetos P., Kurochkina N., Binukumar B.K., Amin N.D., Terse A., Joseph J., Raithel S.J. (2018). Phosphorylation of the Transient Receptor Potential Ankyrin 1 by Cyclin-dependent Kinase 5 affects Chemo-nociception. Sci. Rep..

[127] Falkenburger B.H., Jensen J.B., Hille B. (2010). Kinetics of PIP2 metabolism and KCNQ2/3 channel regulation studied with a voltage-sensitive phosphatase in living cells. J. Gen. Physiol..

[128] Rodríguez-Menchaca A.A., Adney S.K., Zhou L., Logothetis D.E. (2012). Dual Regulation of Voltage-Sensitive Ion Channels by PIP(2). Front. Pharmacol..

[129] Zaydman M.A., Cui J. (2014). PIP2 regulation of KCNQ channels: Biophysical and molecular mechanisms for lipid modulation of voltage-dependent gating. Front. Physiol..

[130] Gandini M.A., Felix R. (2012). Functional interactions between voltage-gated Ca(2+) channels and Rab3-interacting molecules (RIMs): New insights into stimulus-secretion coupling. Biochim. Biophys. Acta.

[131] McMillan H., Lundy F.T., Dunne O.M., Al-Natour B., Jeanneau C., About I., Curtis T.M., El Karim I. (2021). Endogenous Mas-related G-protein-coupled receptor X1 activates and sensitizes TRPA1 in a human model of peripheral nerves. FASEB J..

[132] Wang H., Xu H., Wu L.J., Kim S.S., Chen T., Koga K., Descalzi G., Gong B., Vadakkan K.I., Zhang X. (2011). Identification of an adenylyl cyclase inhibitor for treating neuropathic and inflammatory pain. Sci. Transl. Med..

[133] Kim K., Han Y., Duan L., Chung K.Y. (2022). Scaffolding of Mitogen-Activated Protein Kinase Signaling by β-Arrestins. Int. J. Mol. Sci..

[134] Murthy S.E., Loud M.C., Daou I., Marshall K.L., Schwaller F., Kühnemund J., Francisco A.G., Keenan W.T., Dubin A.E., Lewin G.R. (2018). The mechanosensitive ion channel Piezo2 mediates sensitivity to mechanical pain in mice. Sci. Transl. Med..

[135] Chompunud Na Ayudhya C., Roy S., Thapaliya M., Ali H. (2020). Roles of a Mast Cell-Specific Receptor MRGPRX2 in Host Defense and Inflammation. J. Dent. Res..

[136] Lazki-Hagenbach P., Ali H., Sagi-Eisenberg R. (2021). Authentic and Ectopically Expressed MRGPRX2 Elicit Similar Mechanisms to Stimulate Degranulation of Mast Cells. Cells.

[137] Higashio H., Satoh Y., Saino T. (2016). Mast cell degranulation is negatively regulated by the Munc13-4-binding small-guanosine triphosphatase Rab37. Sci. Rep..

[138] Jimenez-Vargas N.N., Pattison L.A., Zhao P., Lieu T., Latorre R., Jensen D.D., Castro J., Aurelio L., Le G.T., Flynn B. (2018). Protease-activated receptor-2 in endosomes signals persistent pain of irritable bowel syndrome. Proc. Natl. Acad. Sci. USA.

[139] García-Domínguez M. (2025). NGF in Neuropathic Pain: Understanding Its Role and Therapeutic Opportunities. Curr. Issues Mol. Biol..

[140] Schmelz M. (2019). Itch Processing in the Skin. Front. Med..

[141] Lu P., Zhao Y., Xie Z., Zhou H., Dong X., Wu G.F., Kim B.S., Feng J., Hu H. (2023). MrgprA3-expressing pruriceptors drive pruritogen-induced alloknesis through mechanosensitive Piezo2 channel. Cell Rep..

[142] Jiang M., Chen M., Liu N. (2024). Interactions between Schwann cell and extracellular matrix in peripheral nerve regeneration. Front. Neurol..

[143] Nieuwenhuis B., Haenzi B., Andrews M.R., Verhaagen J., Fawcett J.W. (2018). Integrins promote axonal regeneration after injury of the nervous system. Biol. Rev. Camb. Philos. Soc..

[144] Lefcort F., Venstrom K., McDonald J.A., Reichardt L.F. (1992). Regulation of expression of fibronectin and its receptor, alpha 5 beta 1, during development and regeneration of peripheral nerve. Development.

[145] Toma J.S., Karamboulas K., Carr M.J., Kolaj A., Yuzwa S.A., Mahmud N., Storer M.A., Kaplan D.R., Miller F.D. (2020). Peripheral Nerve Single-Cell Analysis Identifies Mesenchymal Ligands that Promote Axonal Growth. eNeuro.

[146] Fagoe N.D., Attwell C.L., Kouwenhoven D., Verhaagen J., Mason M.R. (2015). Overexpression of ATF3 or the combination of ATF3, c-Jun, STAT3 and Smad1 promotes regeneration of the central axon branch of sensory neurons but without synergistic effects. Hum. Mol. Genet..

[147] Deng L., Gillis J.E., Chiu I.M., Kaplan D.H. (2024). Sensory neurons: An integrated component of innate immunity. Immunity.

[148] Qu L., Fan N., Ma C., Wang T., Han L., Fu K., Wang Y., Shimada S.G., Dong X., LaMotte R.H. (2014). Enhanced excitability of MRGPRA3- and MRGPRD-positive nociceptors in a model of inflammatory itch and pain. Brain.

[149] Bourinet E., Altier C., Hildebrand M.E., Trang T., Salter M.W., Zamponi G.W. (2014). Calcium-permeable ion channels in pain signaling. Physiol. Rev..

[150] Tucker W.C., Chapman E.R. (2002). Role of synaptotagmin in Ca2+-triggered exocytosis. Biochem. J..

[151] Tokuoka H., Goda Y. (2003). Synaptotagmin in Ca2+ -dependent exocytosis: Dynamic action in a flash. Neuron.

[152] Xue R., Meng H., Yin J., Xia J., Hu Z., Liu H. (2021). The Role of Calmodulin vs. Synaptotagmin in Exocytosis. Front. Mol. Neurosci..

[153] García-Domínguez M. (2025). Injury-Driven Structural and Molecular Modifications in Nociceptors. Biology.

[154] Fan Y., Kim D.H., Ryu Y., Chang S., Lee B.H., Yang C.H., Kim H.Y. (2018). Neuropeptides SP and CGRP Underlie the Electrical Properties of Acupoints. Front. Neurosci..

[155] Chiu I.M., von Hehn C.A., Woolf C.J. (2012). Neurogenic inflammation and the peripheral nervous system in host defense and immunopathology. Nat. Neurosci..

[156] Todd A.J. (2010). Neuronal circuitry for pain processing in the dorsal horn. Nat. Rev. Neurosci..

[157] Ricoy U.M., Frerking M.E. (2014). Distinct roles for Cav2.1-2.3 in activity-dependent synaptic dynamics. J. Neurophysiol..

[158] Dolphin A.C. (2021). Functions of Presynaptic Voltage-gated Calcium Channels. Function.

[159] Zhang W., Jiang H.H., Luo F. (2022). Diverse organization of voltage-gated calcium channels at presynaptic active zones. Front. Synaptic Neurosci..

[160] D’Mello R., Dickenson A.H. (2008). Spinal cord mechanisms of pain. Br. J. Anaesth..

[161] Woolf C.J. (2022). Pain modulation in the spinal cord. Front. Pain Res..

[162] Boehm J., Malinow R. (2005). AMPA receptor phosphorylation during synaptic plasticity. Biochem. Soc. Trans..

[163] Jenkins M.A., Traynelis S.F. (2012). PKC phosphorylates GluA1-Ser831 to enhance AMPA receptor conductance. Channels.

[164] Sala C., Segal M. (2014). Dendritic spines: The locus of structural and functional plasticity. Physiol. Rev..

[165] Nakahata Y., Yasuda R. (2018). Plasticity of Spine Structure: Local Signaling, Translation and Cytoskeletal Reorganization. Front. Synaptic Neurosci..

[166] Gong H., Xu X., Talifu Z., Zhang C.J., Sun Y.Z., Yue Z.M., Rao J.S., Du L.J., Du X.X. (2025). Prospects and challenges in NMDAR signaling in spinal cord injury recovery and neural circuit remodeling. Regen. Ther..

[167] Kano M., Ohno-Shosaku T., Hashimotodani Y., Uchigashima M., Watanabe M. (2009). Endocannabinoid-mediated control of synaptic transmission. Physiol. Rev..

[168] García-Domínguez M. (2025). Role of the Endocannabinoid System in Fibromyalgia. Curr. Issues Mol. Biol..

[169] Yadav R., Yan X., Maixner D.W., Gao M., Weng H.R. (2015). Blocking the GABA transporter GAT-1 ameliorates spinal GABAergic disinhibition and neuropathic pain induced by paclitaxel. J. Neurochem..

[170] Mapplebeck J.C.S., Lorenzo L.E., Lee K.Y., Gauthier C., Muley M.M., De Koninck Y., Prescott S.A., Salter M.W. (2019). Chloride Dysregulation through Downregulation of KCC2 Mediates Neuropathic Pain in Both Sexes. Cell Rep..

[171] Imlach W.L., Bhola R.F., Mohammadi S.A., Christie M.J. (2016). Glycinergic dysfunction in a subpopulation of dorsal horn interneurons in a rat model of neuropathic pain. Sci. Rep..

[172] Azimi E., Reddy V.B., Shade K.C., Anthony R.M., Talbot S., Pereira P.J.S., Lerner E.A. (2016). Dual action of neurokinin-1 antagonists on Mas-related GPCRs. JCI Insight.

[173] Bawazir M., Amponnawarat A., Hui Y., Oskeritzian C.A., Ali H. (2022). Inhibition of MRGPRX2 but not FcεRI or MrgprB2-mediated mast cell degranulation by a small molecule inverse receptor agonist. Front. Immunol..

[174] Macphee C.H., Dong X., Peng Q., Paone D.V., Skov P.S., Baumann K., Roethke T., Goldspink D.A., Pearson S.K., Wu Z. (2024). Pharmacological blockade of the mast cell MRGPRX2 receptor supports investigation of its relevance in skin disorders. Front. Immunol..

[175] Kumar M., Duraisamy K., Annapureddy R.R., Chan C.B., Chow B.K.C. (2023). Novel small molecule MRGPRX2 antagonists inhibit a murine model of allergic reaction. J. Allergy Clin. Immunol..

[176] Sanjel B., Teli M.K., Kumar S., Oh J.H., Maeng H.J., Shim W.S., Kim M.H. (2025). Identification of KMH-45, a novel MRGPRX2 inhibitor with enhanced anti-pruritic properties. Biomed. Pharmacother..

[177] Dando T.M., Perry C.M. (2004). Aprepitant: A review of its use in the prevention of chemotherapy-induced nausea and vomiting. Drugs.

[178] Chain A., Wrishko R., Vasilinin G., Mouksassi S. (2020). Modeling and simulation analysis of aprepitant pharmacokinetics in pediatric patients with postoperative or chemotherapy-induced nausea and vomiting. J. Pediatr. Pharmacol. Ther..

[179] Curran P.M., Robinson D.M. (2009). Aprepitant: A review of its use in the prevention of nausea and vomiting. Drugs.

[180] Santini D., Vincenzi B., Guida F.M., Imperatori M., Schiavon G., Venditti O., Frezza A.M., Berti P., Tonini G. (2012). Aprepitant for management of severe pruritus related to biological cancer treatments: A pilot study. Lancet Oncol..

[181] Zhou T., Zhang Y., Ma Y., Ma W., Wu X., Huang L., Feng W., Zhou H., Liu J., Zhao H. (2022). Comparison of aprepitant versus desloratadine for EGFR-TKI-induced pruritus: A randomized phase 2 clinical trial. Cancer.

[182] Le Gall-Ianotto C., Verdet R., Nowak E., Le Roux L., Gasse A., Fiedler A., Carlhant-Kowalski D., Marcorelles P., Misery L., Ianotto J.C. (2021). Rationale and design of the multicentric, double-blind, double-placebo, randomized trial APrepitant versus HYdroxyzine in association with cytoreductive treatments for patients with myeloproliferative neoplasia suffering from persistent aquagenic pruritus. Trial acronym: APHYPAP. Trials.

[183] Ohanyan T., Schoepke N., Eirefelt S., Hoey G., Koopmann W., Hawro T., Maurer M., Metz M. (2018). Role of substance P and its receptor neurokinin 1 in chronic prurigo: A randomized, proof-of-concept, controlled trial with topical aprepitant. Acta Derm. Venereol..

[184] Zic J.A., Straka B.T., McGirt L.Y., Nian H., Yu C., Brown N.J. (2018). Aprepitant for the treatment of pruritus in Sézary syndrome: A randomized crossover clinical trial. JAMA Dermatol..

[185] McNeil B.D. (2021). MRGPRX2 and Adverse Drug Reactions. Front. Immunol..

[186] Ogasawara H., Noguchi M. (2021). Therapeutic Potential of MRGPRX2 Inhibitors on Mast Cells. Cells.

